# Dynamics of tRNA fragments and their targets in aging mammalian brain

**DOI:** 10.12688/f1000research.10116.1

**Published:** 2016-11-24

**Authors:** Spyros Karaiskos, Andrey Grigoriev

**Affiliations:** 1 Department of Biology, Center for Computational and Integrative Biology, Rutgers University, Camden, USA

**Keywords:** transfer RNA, rat brain, rat cortex, tRNA fragments, aging, non-coding RNA

## Abstract

*Background: *The progress of next-generation sequencing technologies has unveiled various non-coding RNAs that have previously been considered products of random degradation and attracted only minimal interest. Among small RNA families, microRNA (miRNAs) have traditionally been considered key post-transcriptional regulators. However, recent studies have reported evidence for widespread presence of fragments of tRNA molecules (tRFs) across a range of organisms and tissues, and of tRF involvement in Argonaute complexes. *Methods:*To elucidate potential tRF functionality, we compared available RNA sequencing datasets derived from the brains of young, mid-aged and old rats. Using sliding 7-mer windows along a tRF, we searched for putative seed sequences with high numbers of conserved complementary sites within 3' UTRs of 23 vertebrate genomes. We analyzed Gene Ontology term enrichment of predicted tRF targets and compared their transcript levels with targets of miRNAs in the context of age. *Results and Discussion: *We detected tRFs originating from 3’- and 5’-ends of tRNAs in rat brains at significant levels. These fragments showed dynamic changes: 3’ tRFs monotonously increased with age, while 5’ tRFs displayed less consistent patterns. Furthermore, 3’ tRFs showed a narrow size range compared to 5’ tRFs, suggesting a difference in their biogenesis mechanisms. Similar to our earlier results in *Drosophila *and compatible with other experimental findings, we found “seed” sequence locations on both ends of different tRFs. Putative targets of these fragments were found to be enriched in neuronal and developmental functions. Comparison of tRFs and miRNAs increasing in abundance with age revealed small, but distinct changes in brain target transcript levels for these two types of small RNA, with the higher proportion of tRF targets decreasing with age. We also illustrated the utility of tRF analysis for annotating tRNA genes in sequenced genomes.

## Introduction

Small RNA molecules derived from fragmented tRNAs form a new class of short (~16–40 nt) RNA molecules. They arise from directed cleavage of cellular tRNAs, including both tRNA precursor species, as well as mature, functional tRNA molecules, and have been associated with multiple infectious diseases, pathogen resistance and regulation ^1, 2^. Early reports described such fragments resulting from cleavage of tRNAs in *Escherichia coli* as a protective response to phage infection and as “biochemical warfare” directed against unrelated bacterial strains ^3, 4^. Subsequent studies have expanded the known domain of these fragments to archaea ^5^, eukaryotes ^6– 8^, including their parasites ^9, 10^, and to human cells ^7, 11– 14^. Broadly, the fragments are categorized into two types based on length and biogenesis: tRNA halves and tRNA-derived fragments (tRFs); this paper is focused on the latter. Studied and reviewed by several experimental groups ^15– 18^, tRFs are molecules of ~16–24 nt in length and can be classified into three types based on the tRNA region from which they derive: 5' tRF, 3'CCA and 3'U tRF. The last two types originate from the 3’ end of the tRNA, while the first is derived from the 5’ end. The 3'CCA type is generated from the 3' end of the mature tRNA and includes the CCA that is added to all tRNAs post-transcriptionally. The 3'U type is derived from the uracil rich trailer sequence upstream of the 3' end of the precursor tRNA molecule and has multiple Us added to the 3’ end. There have been various attempts to determine the biogenesis and function of these different types of tRNA-derived small RNAs, but currently most of these questions are still open.

Hypothesized to function similarly to microRNAs (miRNAs), either by regulating mRNAs (like miRNAs) or by affecting miRNA loading and processing ^7, 11, 19^, tRFs have also been shown to bind to Argonaute complexes in multiple species ^20, 21^, strengthening their likely role in RISC-mediated gene silencing. A meta-analysis of PAR-CLIP libraries found that both 5’ and 3’ CCA tRFs were loaded to Ago1, Ago3, and Ago4, but 3’ U tRFs did not associate with Argonaute proteins in great numbers in human cells ^21^. A recent study suggested a traditional miRNA-like silencing based on complementarity of the 5' seed sequence of a tRF to a sub-sequence within a 3' UTR of a transcript ^19^. Yet another study has shown that the last 8–10 nts on the 3’ end of the tRF are responsible for mRNA repression ^22^. In our lab, using a computational approach similar to detection of miRNA seeds, we have found potential seed regions on both a 5'-and a 3' tRF end ^23^. Adding to this similarity, we have also reported age-related changes of tRF abundance in *Drosophila melanogaster* ^23^, comparable to those detected for miRNA in the same organism ^20^. Such changes with age were also detected in tRFs of *Caenorhabditis elegans* ^24^.

Here, we report on further support for such miRNA likeness of tRFs in another experimental system, which shows that both of these types of small RNA may participate in the mechanisms of brain aging. Aging underlies cognitive decline and dementia, and is the greatest risk factor for the failure of brain functioning in adults. Analysis of aging brain can shed light on the basic neurological mechanisms and their connections with age-related neurodegenerative conditions, such as Alzheimer's and Parkinson's disease ^25^. Neurological research has used rats extensively over many years as models for mammalian behavioral and neurodegeneration studies. In the present study, we analyzed available RNA sequencing libraries produced from the brains of rats of different ages ^26^ and identified numerous tRFs, which showed consistent changes in their abundance patterns with age. We also confirmed in rat brains our previous findings on possible targeting mode of *Drosophila* tRFs and the functional enrichment of their targets in neuronal and developmental functions ^23^. Potential targets of tRFs with clearly defined seeds showed higher levels of down-regulation with age compared to the rest of the brain transcriptome and to the targets of miRNAs upregulated with age. Our results strengthen the emerging consensus that tRFs are a novel class of non-coding RNA molecules; they target mRNAs in a manner similar to miRNAs and their abundance in the cell is dynamically regulated with regards to aging.

## Methods

### Small RNA analysis

We used small RNA sequencing libraries from brains of the rat *Rattus norvegicus* ^26^ publicly available from the European Nucleotide Archive (accession number, ERA365111). Using the sra-toolkit 2.8.0 ( https://trace.ncbi.nlm.nih.gov/Traces/sra/sra.cgi?view=software) we converted the sra files to fastq format. We used fastx toolkit 0.0.13 ( http://hannonlab.cshl.edu/fastx_toolkit/) to clip the adapter sequences and collapse identical reads. The reads of length above 16 nts were used for downstream analysis. We collapsed and mapped the reads to the rat genome (rn6, http://hgdownload.soe.ucsc.edu/goldenPath/rn6/bigZips/), and also to the union of rat tRNAs from two independent databases ( http://gtrnadb.ucsc.edu and http://trnadb.bioinf.uni-leipzig.de, also including mitochondrial tRNA genes from the latter) using Bowtie (version 1.1.1, released on 10/1/2014, http://bowtie-bio.sourceforge.net/index.shtml). Bowtie parameters were set to output only perfect matches to tRNA sequences, including the post transcriptional CCA modification. Read counts in each experiment were normalized by the total number of reads detected, and averaged across three replicates for each of the three time points (ages of 6, 14 and 22 months). For further analysis, we selected only tRFs with read counts >0.1% of total reads in every replicate.

### Seed sequence analysis

We generated 7-mer subsequences of tRFs by applying a 7-nt sliding window and shifting by one nt from the 5’ to the 3’ end. We then found the counts of exact matches for each of these subsequences to the 3’ UTR regions conserved in at least 15 species (always including human, mouse and rat) out of 23 ( Table 1; alignments obtained from http://www.targetscan.org/). To estimate significance of the seed matches, we compared the observed match counts for each respective 7-mer in a tRF to (i) the expected number of matches by chance (estimated from 7-mer genomic frequency) and (ii) the average number of matches of all possible 7-mers with the same nucleotide composition in conserved 3’UTRs. Genes with exact matches of 7-mer and 7-mer_1a candidate seeds to the 3’UTR were considered potential targets.

**Table 1.** Vertebrate species used for finding seed sequence matches. In total, 23 species are listed with their taxonomy IDs. Matches in at least 15 of these species were required, always including human, mouse and rat (shaded rows).

Taxonomy ID	Species
10090	Mus musculus
10116	Rattus norvegicus
10141	Cavia porcellus
13616	Monodelphis domestica
28377	Anolis carolinensis
30611	Otolemur garnettii
37347	Tupaia belangeri
42254	Sorex araneus
8364	Xenopus tropicalis
9031	Gallus gallus
9258	Ornithorhynchus anatinus
9361	Dasypus novemcinctus
9365	Erinaceus europaeus
9371	Echinops telfairi
9544	Macaca mulatta
9598	Pan troglodytes
9606	Homo sapiens
9615	Canis lupus familiaris
9685	Felis catus
9785	Loxodonta africana
9796	Equus caballus
9913	Bos taurus
9986	Oryctolagus cuniculus

### RNA sequencing analysis

For target expression analysis, we downloaded files with pre-computed transcript expression levels for the rat cerebral cortex transcriptome (GEO data series; accession number, GSE34272 ^27^). The expression levels in each experiment were normalized by the total number of reads detected and averaged across three replicates for each of the three time points (ages of 6, 12 and 28 months).

### Statistical evaluation of downregulation levels for miRNA and tRF targets

For each set of predicted targets of a tRF or miRNA, we compared its ratio of down-regulated/up-regulated target transcripts from young to old rats with the distribution of such ratios calculated for 1,000 randomly selected transcript sets (from the same transcriptome) of the same size as the target set (different for each tRF and miRNA). This process was repeated for three different thresholds (up-regulated by >5% / downregulated by >5%; up-regulated by >10% / downregulated by >10%; and up-regulated by >20% / downregulated by >20%), and the statistical significance of the differences observed was obtained using two-tailed t-test in the R package (version 3.3.1; www.R-project.org).

### Gene ontology enrichment analysis

The predicted targets for each tRF were used as input in order to perform GO enrichment analysis. Each set of targets was uploaded to PANTHER website ( http://pantherdb.org/; version 11.1 ^28^) and results were obtained using the website default parameters.

## Results and discussion

### tRNA fragments in rat brain

We analyzed available datasets of nine different small-RNA libraries corresponding to three replicates for three distinct time points throughout a rat lifespan. These libraries were originally produced to study miRNA in the brains of young, middle-aged and old rats ^26^. We will refer to the results associated with these three time points (6, 14 and 22 months) as Y, M and O, respectively. After mapping short RNA reads from these libraries to the union of tRNA sequences obtained from two independent databases ( http://gtrnadb.ucsc.edu; http://trna.bioinf.uni-leipzig.de), we observed that the vast majority of alignments localized preferentially to a 5'- or a 3'-end of a tRNA molecule. Only 1–7% of the reads among the nine sequencing experiments aligned elsewhere on the tRNA sequence. In the datasets we analyzed, a negligible number of reads aligned to 3' U tRFs, therefore we limited our focus to 5' and 3' tRFs, for which there was extensive evidence. The two dominant tRF classes appeared likely to be generated by different mechanisms of cleavage. For instance, there was a striking consistency regarding the cleavage site location in 3’ tRFs, compared to a wider distribution of those sites in 5' tRFs ( Figure 2), supporting the notion that tRFs are not byproducts of random degradation, but have specific structure-dependent cleavage sites.

**Figure 1.**
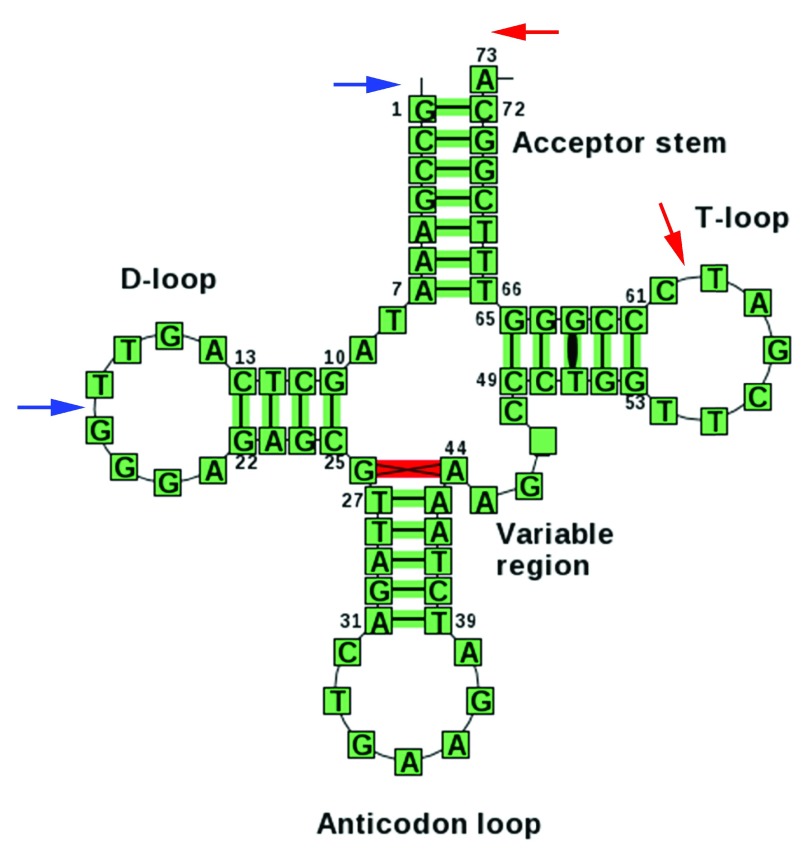
Typical endpoints of tRNA fragments. Typical secondary structure representation for the PheGAA tRNA gene (from http://trnadb.bioinf.uni-leipzig.de). Blue arrows point to typical endpoints for a 5' tRF. Red arrows indicate the ends of the most frequent 3' tRF. The mature tRNA molecule also contains the post-transcriptional 3' CCA modification (as does the 3' tRF). A 3' U tRF would derive from the uracil-rich trailer sequence downstream of the end of the tRNA gene (not shown).

**Figure 2.**
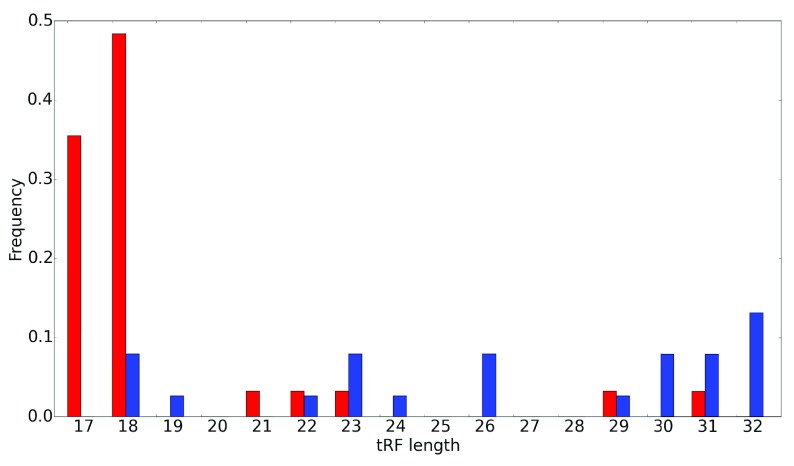
Differences in length distribution of tRFs. Length distributions for 5' (blue) and 3' (red) tRFs. tRF length is shown on the x-axis and the frequency on the y-axis. Note the much broader variability for the 5’ tRFs.

### Age-related patterns of tRF abundance

**Figure 3.**
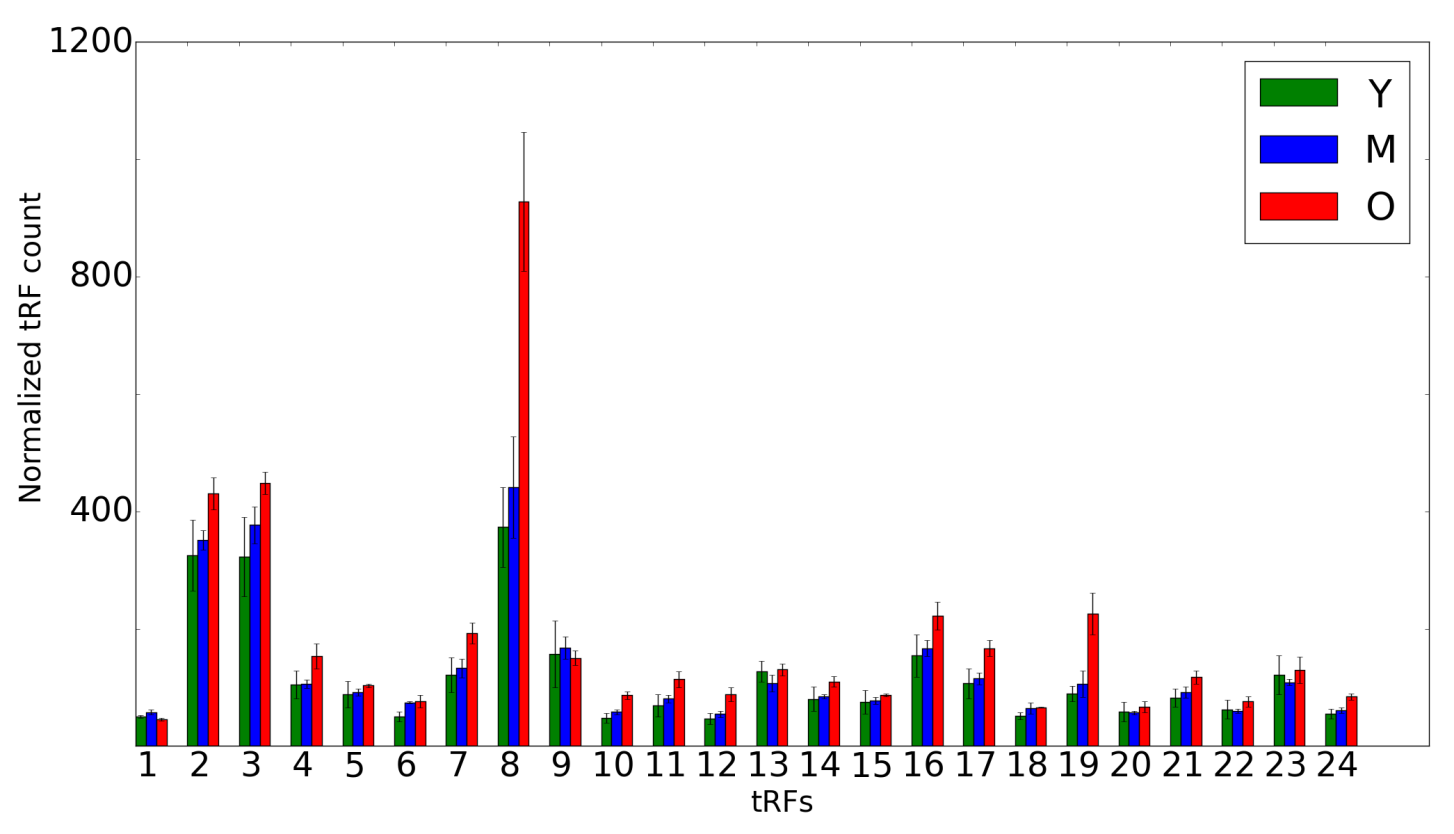
Age-related change in 3' tRF levels. Abundance of 3' tRFs in rat brains for 3 distinct time points (Y is shown in green, M in blue and O in red). An average of 3 replicates for each tRF is shown on the x-axis for each time point. Error bars indicate the range of read counts. The y-axis shows the normalized tRF abundance; the numbers on the x-axis correspond to tRNA genes listed in Table 2. Y, young (6 months); M, mid-age (14 months); O, old (22 months).

**Figure 4.**
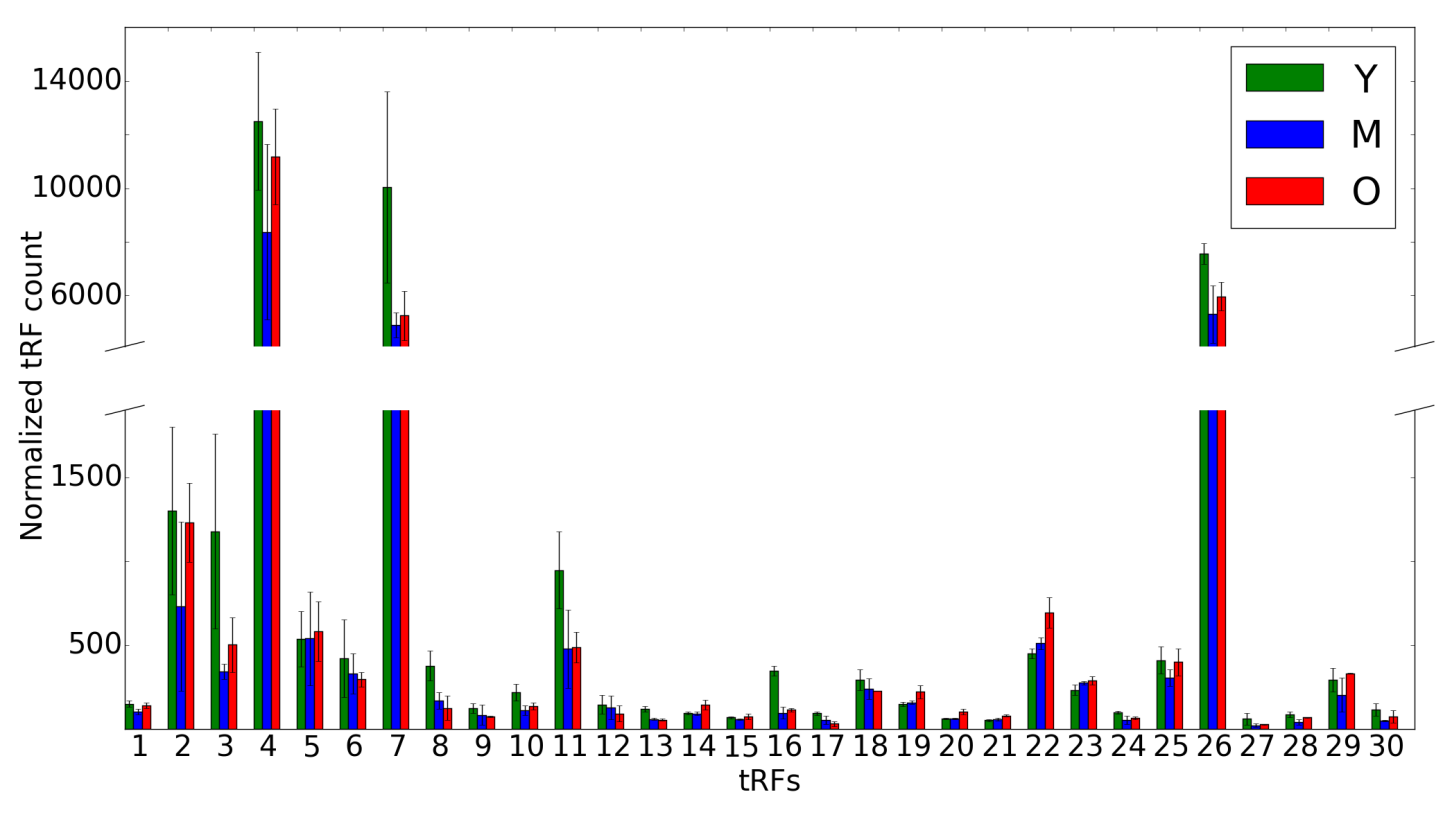
Age-related change in 5' tRF levels. Abundance of 5' tRFs in rat brains for 3 distinct time points (Y is shown in green, M in blue and O in red). An average of 3 replicates for each tRF is shown on the x-axis for each time point. Error bars indicate the range of read counts. The y-axis shows the normalized tRF abundance; the numbers on the x axis correspond to tRNA genes listed in Table 2. Y, young (6 months); M, mid-age (14 months); O, old (22 months).

**Figure 5.**
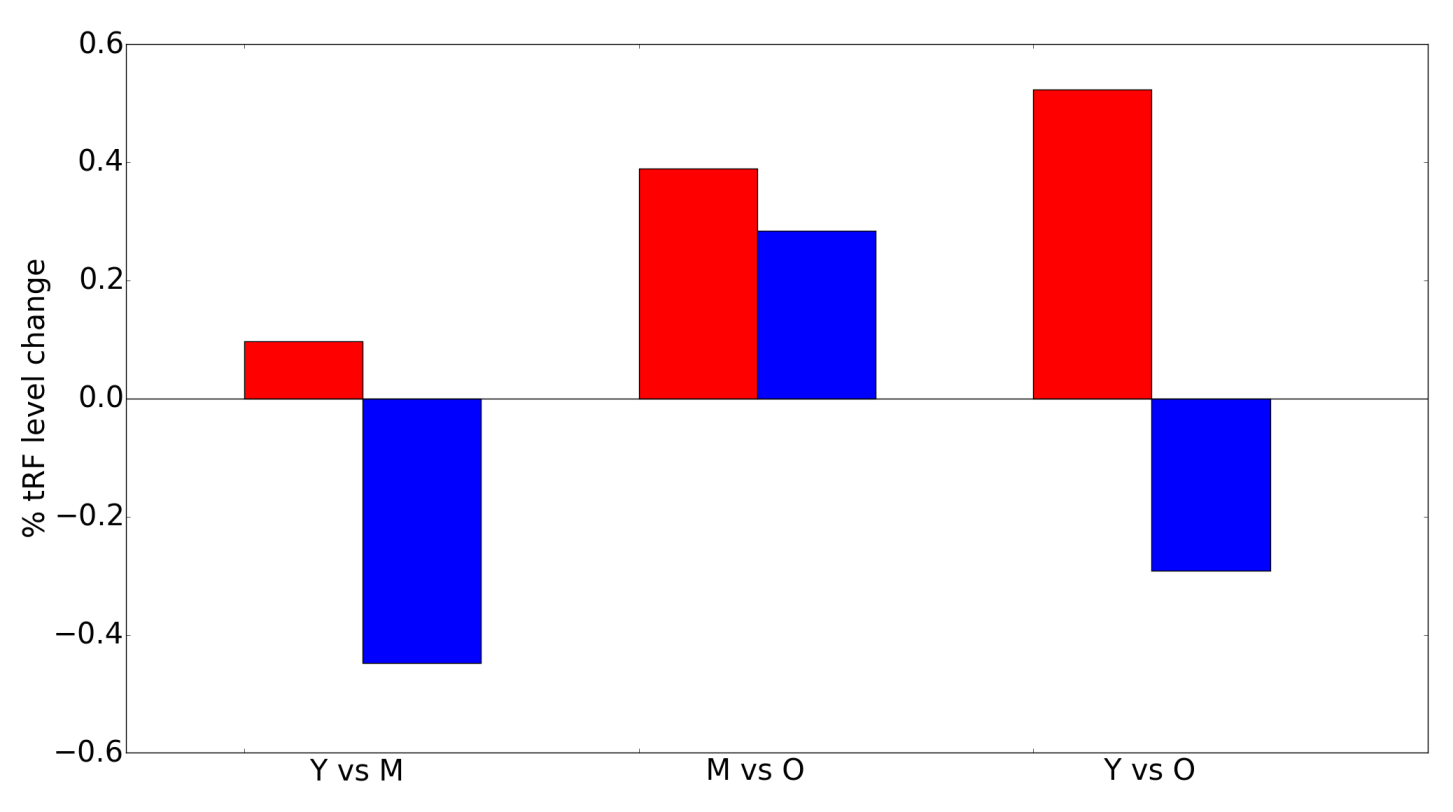
Total tRF abundance. Change in total abundance levels with age for all 5' tRFs (blue) and 3' tRFs (red). Y, young (6 months); M, mid-age (14 months); O, old (22 months).

We then analyzed age-related abundance of 3' and 5’ tRFs in the brain. Interestingly, we observed a very common trend of an overall monotonous increase in the 3' tRF levels with age, Y < M < O ( Figure 3; Table 2). In striking contrast, the 5’ tRFs displayed a much less consistent picture ( Figure 4; Table 2), with several cases of monotonous increase or decrease with age, but mostly with a visibly different pattern of change M < Y < O ( Figure 5). This difference, together with the cleavage site distributions ( Figure 2), suggests that distinct processes are likely responsible for the generation of 3’ and 5’ tRFs, which may be relevant for their function.

**Table 2.** tRFs shown in Figure 3 and Figure 4. The tRNA anticodon is shown in column 1, the tRNA ID in column 2 and the corresponding numbers used as an x axis label for Figure 3 and Figure 4 (when applicable) are shown in columns 3 and 4, respectively. A database, which each matching tRNA gene was downloaded from, is shown in column 5: * http://gtrnadb.ucsc.edu; ** http://trnadb.bioinf.uni-leipzig.de. Additionally, a dollar sign ($) in column 5, indicates that a potential seed was detected in this tRF. Coordinates of tRFs on the matching tRNA genes are given in column 6.

tRNA Anticodon	tRNA ID	Figure 3 (3' tRFs)	Figure 4 (5' tRFs)	Database	tRF Coordinates on tRNA molecule (3', 5' when both are shown)
TrpTCA	MtdbD00003370	1	--	** (mitochondrial)	49-69
ValCAC	trna2253	2	25	*	59-76, 1-32
ValAAC	trna1605	3	--	*, $	60-76
TrpCCA	trna11440	4	--	*	58-75
LeuAAG	trna6448	5	--	*	68-85
LeuCAG	trna3830	6	--	*	69-86
SerGCT	trna9151	7	--	*, $	68-85
ProTGG	trna13310	8	--	*, $	59-75
SerAGA	trna455	9	20	*, $	68-85
SerTGA	trna2516	10	--	*	68-85
GlnCTG	trna1868	11	--	*	58-75
GlnCTG	trna1911	12	--	*	58-75
ValTAC	MtdbD00003196	13	--	** (mitochondrial)	49-71
LysCTT	trna186	14	--	*	60-76
PheGAA	trna3690	15	--	*, $	60-76
ArgACG	trna1588	16	--	*	59-76
TrpCCA	trna2467	17	--	*	59-75
AlaTGC	trna5057	18	14	*, $	58-75, 1-22
ProAGG	trna13311	19	--	*	58-75
GlyGCC	trna1527	20	5	*	58-74, 1-30
ArgACG	trna1600	21	--	*	60-76
GlyTCC	trna2250	22	--	*	59-75
GlyCCC	trna7585	23	--	*	57-74
GlyCCC	trna2752	24	--	*	57-74
IniCAT	MtdbD00003481	--	1	** (mitochondrial)	1-30
GlyGCC	trna2377	--	2	*	1-18
GlyGCC	trna2376	--	3	*	1-32
GlyGCC	trna3490	--	4	*	1-18
GlyGCC	trna1897	--	6	*	1-31
GlyGCC	trna1528	--	7	*	1-32
GlyGCC	trna11254	--	8	*	1-34
LysCTT	trna7564	--	9	*	1-26
LysCTT	trna2975	--	10	*	1-33
LysCTT	trna13312	--	11	*	1-34
HisGTG	trna3497	--	12	*	1-30
HisGTG	trna4377	--	13	*	1-33
AlaTGC	trna12492	--	15	*	1-23
CysGCA	trna8932	--	16	*	1-32
CysGCA	trna8966	--	17	*	1-33
TyrGTA	MtdbD00003620	--	18	** (mitochondrial)	1-31
SerAGA	trna3680	--	19	*	1-23
LeuCAG	trna2365	--	21	*, $	1-18
LeuCAG	trna2370	--	22	*	1-26
ValCAC	trna1607	--	23	*	1-23
ValAAC	trna3670	--	24	*	1-33
ValCAC	trna1601	--	26	*	1-33
ValCAC	trna4363	--	27	*	1-36
GlnTTG	trna4	--	28	*	1-36
GluCTC	tdbD00000658	--	29	**, $	1-19
GluCTC	trna2251	--	30	*	1-34

### Computational prediction of tRF seeds and their targets

Given the significant levels of tRFs in rat brains and their dynamic changes with age, we aimed to investigate their possible effect on the brain transcriptome. Although the mechanism of tRF action is yet to be elucidated, there is recent evidence suggesting an animal miRNA-like pathway of action. Previous reports have detected tRNA fragments in the cytoplasmic fraction of various human cells, including B-cells and A549 cells ^22, 29^, as well as mouse ES cells, plant cells, fission yeast cells and carcinoma cell lines, including HepG2, LNCap and LNCap-derived C4-2 ^13, 30– 33^. It has been proposed that tRFs are likely to function similarly to a traditional miRNA-like mode, using perfect complementarity of the 5' seed sequence of the tRF (typically, positions 2–8 in miRNAs) to target a subsequence within a 3' UTR of a transcript ^29^. Contrary to the above, an alternative mode of action for tRFs has been suggested by a study ^22^, which utilized luciferase reporter assays to demonstrate that a potential seed sequence resided in the 3' end of the tRNA fragment, ruling out a 5' and a middle segment seed binding.

**Figure 6.**
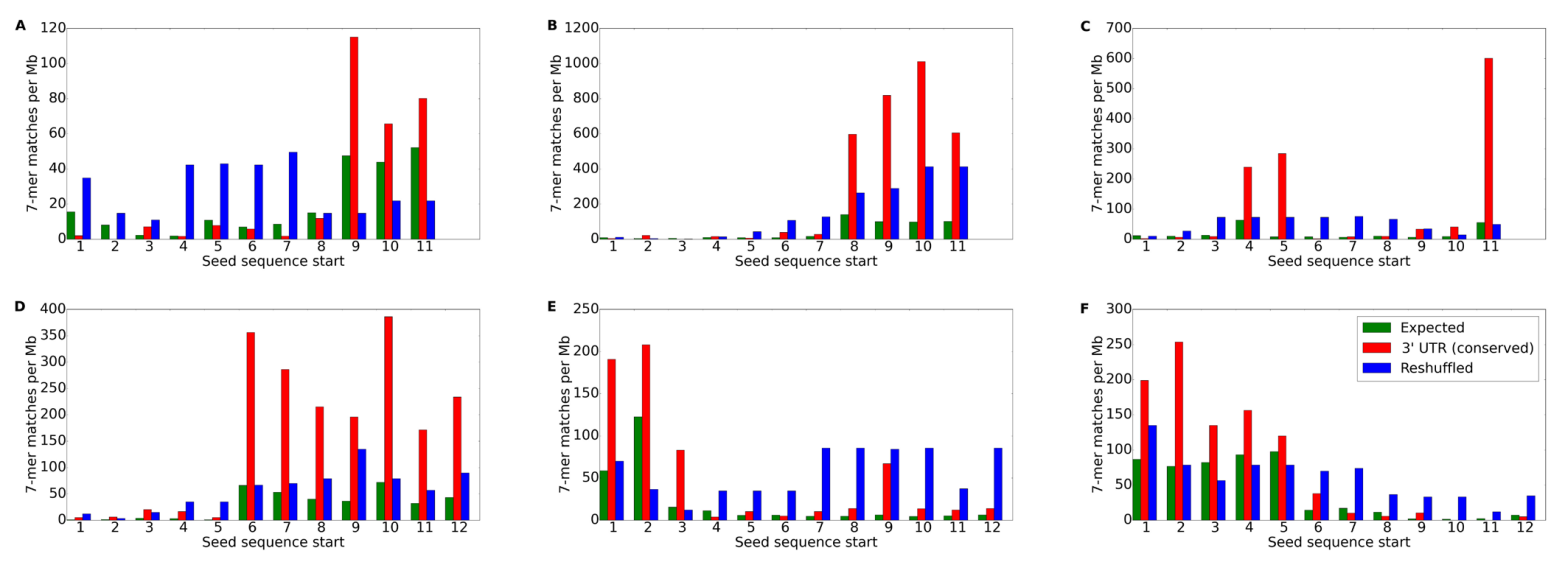
Candidate seed region locations for tRFs. The numbers of exact sequence matches in the 3’ UTR regions are plotted against the starting position of a 7-mer. Expected number of matches in 3' UTRs is shown in yellow, average number of conserved matches for all other 7-mers with the same nucleotide composition as the given window is shown in blue, and the observed number of matches in the conserved regions of 23 vertebrates is shown in red. The letters on the top left corners of each plot correspond to individual tRFs: ( **A**) ProTGG, ( **B**) ValAAC, ( **C**) PheGAA, ( **D**) AlaTGC, ( **E**) SerAGA and ( **F**) SerGCT.

A search for a near-perfect complementarity of tRF sequences against transcripts yielded very few results, both in the 12 *Drosophila* genomes ^23^ and in the present study, further suggesting a targeting mode similar to animal miRNAs. Assuming such an animal-like miRNA targeting mechanism for tRFs, we further investigated the targeting mechanism of tRFs and adjusted our computational pipeline, used previously to find targets in 12 *Drosophila* genomes ^23^, to perform the tRF seed search in mammalian genomes. This pipeline functions similarly to the approach used to identify such seed sequences for miRNAs ^34^. We used 7-nt sliding windows across the length of a tRF sequence and aligned them against conserved 3' UTR regions of 23 vertebrate species. The region was considered conserved if it was found in 15 genomes (always including rat, mouse and human) out of these 23 ( Table 1). We took into consideration the following match types: 7-mer-m8 (full 7-mer match), 7-mer-1a (perfect match of the first 6 nts followed by an A in the 3' end of the targeted transcript) and 8-mer-1a (perfect 7-mer match followed by an A in the 3' end), which have been extensively confirmed for miRNAs in the past ^35^. Our results in finding seeds ( Table 2) demonstrate that such conserved matches can be located both on the 5' end and on the 3' end of the tRF ( Figure 6), concordant with the existing experimentally validated results for tRF targeting mechanisms ^22, 29^. A similar arrangement of the seed regions on the 5' end and on the 3' end of the tRF has also been observed in *Drosophila* ^23^.

The success in finding seeds ( Table 2) was overwhelmingly in favor of shorter 3’ tRFs (6 out of 24, 25%) compared to longer 5’ tRFs (2 out of 30, 6.67%). Given the number of differences between these two tRF types, we chose to focus on 3’ tRFs for the remainder of this paper. For our meta-analysis, detailed in the sections below, we combined experimental results performed in different labs, with different brain material, and at different ages (e.g., 22 months in small RNA-seq series is quite far from both 12 and 28 months in RNA-seq series). Given the small changes in gene expression and to avoid the effects of non-monotonous changes in many 5’tRFs, we limited our subsequent analysis to six 3’ tRFs ( Figure 6), which showed monotonous changes in their levels from Y to M to O and clearly defined seed sequences.

### Gene ontology enrichment analysis of conserved predicted targets

Following our seed region identification for tRFs, we focused on their predicted targets with conserved seed matches within their 3' UTR ( Supplementary File 1). We explored potential functions of targets of six tRFs that showed clearly defined seed sequences ( Figure 6). Gene Ontology (GO) enrichment analysis of conserved predicted targets of these tRFs revealed >150 significantly enriched GO terms for biological process. “Nervous system development” was found to be consistently enriched for all six tRFs, except ProTGG. Additional biological process GO terms, such as “central nervous system development”, “neurogenesis” and “axonogenesis”, were also enriched for multiple tRF targets ( Supplementary File 2). Furthermore, the same tRF targets that showed an enrichment for nervous system functionality and development were also associated with significantly enriched neuron/axon-related cellular localization terms ( Supplementary File 2). Overall, these results are in agreement with our previous work on *D. melanogaster* ^23^, where we have noted a similar enrichment for biological processes related to neuronal function and development for predicted targets of tRFs increasing with age from young to adult flies. However, in addition to these functions, ProTGG and other tRFs also appeared to target transcription and splicing regulators in rat brains ( Supplementary File 1 and Supplementary File 2).

### Expression patterns of predicted tRF targets

We compared our observations of tRF abundance changes with age to the measured expression levels of their targets. We compared the profiles of all mRNAs in the rat cerebral cortex transcriptome ^27^ with those predicted to be targeted by miRNAs (using Targetscan ^34^) and by tRFs (using perfect matching of the identified tRF seed sequence and a conserved target sequence located in the 3' UTR of a transcript). We calculated the ratios of down- to up-regulated transcripts for the whole rat cortex transcriptome and for the targets of six 3’ tRFs (in which seeds could be clearly seen, Figure 6) and five miRNAs ( Table 3) that had >500 raw reads in the old age, and, similarly to 3' tRFs, showed a monotonous increase Y < M < O. We observed that both tRF and miRNA targets were significantly enriched for down-regulated transcripts at three different regulation thresholds ( Table 3). Interestingly, the enrichment for down-regulation in the union set of all neuron-related tRF targets was also significant (p<0.05) for each of these three thresholds of regulation.

**Table 3.** Age-related down-regulation of tRF/miRNA targets. Ratios of down-regulated/up-regulated miRNA- and tRF-targeted transcripts for each of the change thresholds (>5%, >10%, >20%). Significant difference from the expected ratio is indicated by ***p<0.005; **p<0.01; *p<0.05; #p>0.05.

tRF	Ratio (>5%)	Ratio (>10%)	Ratio (>20%)
ProTGG	11.0 ***	7.0 ***	4.0 **
AlaTGC	2.15 *	2.57 **	0.5 *
PheGAA	3.33 ***	3.7 ***	3.25 ***
SerAGA	4.0 ***	4.0 ***	3.0 *
SerGCT	4.0 ***	3.5 ***	2 *
ValAAC	4.1 ***	3.5 ***	1.5 #
miRNA	Ratio (>5%)	Ratio (>10%)	Ratio (>20%)
mir-146b	2.85 ***	2.46 **	4.25 ***
mir-132	2.31 ***	2.24 **	1.56 *
mir-128-2	2.52 ***	2.3 ***	2.5 ***
mir-92a	2.63 ***	2.43 ***	1.89 **
mir-200c	2.71 ***	2.96 ***	1.96 ***

**Figure 7.**
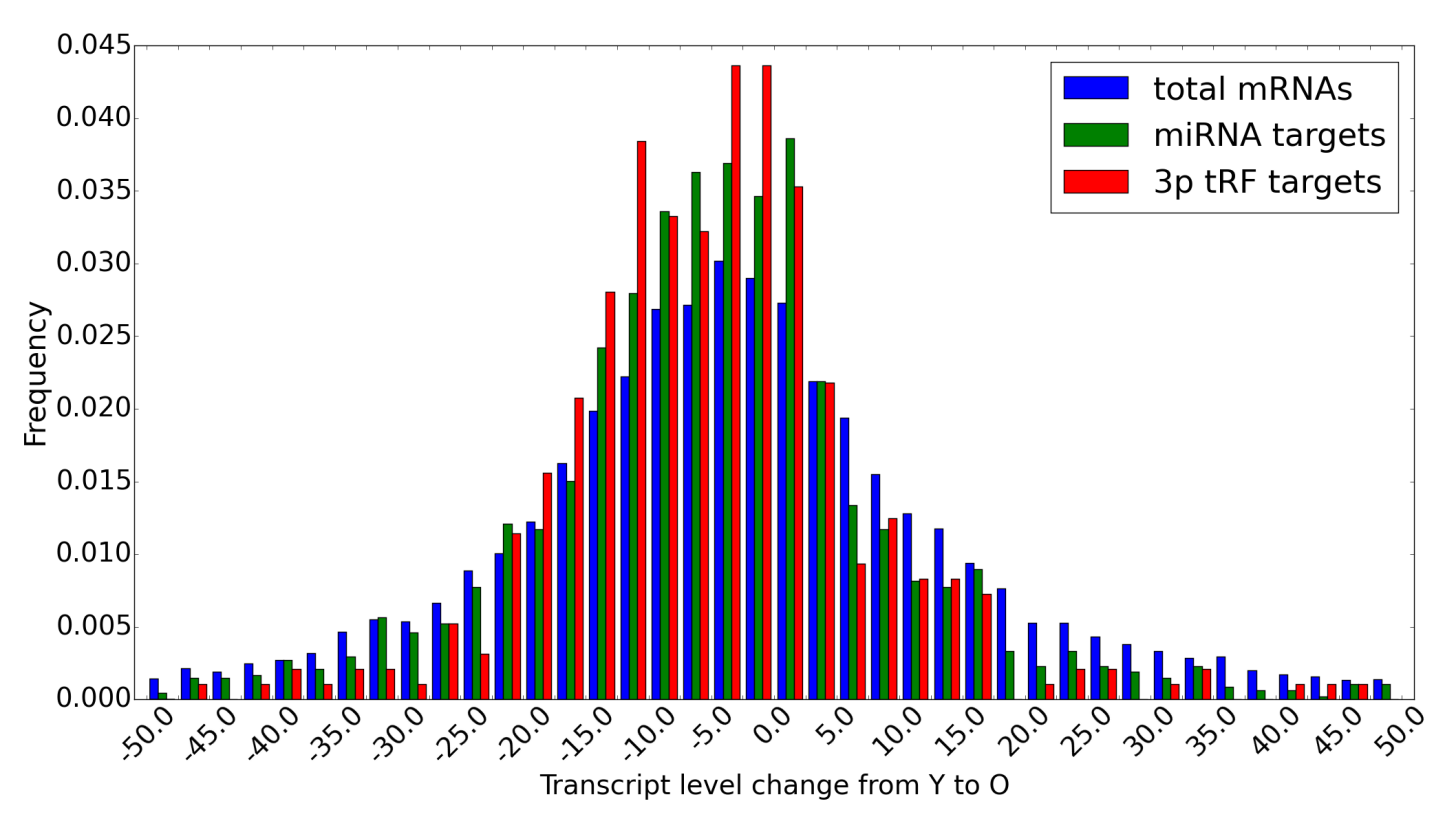
Changes in target transcript levels with age. Transcript level changes from young to old rat brains. Distributions of changes for all detectable mRNAs in rat brains (blue), miRNA-targeted (green) and 3' tRF-targeted transcripts (red) are shown using 2.5% bins.

Comparing the distributions of de-regulation levels from young to old age for (i) all mRNAs detected in rat cortex, (ii) for miRNA-targeted mRNAs and (iii) for tRF targets ( Figure 7), we observed a consistently higher proportion of down-regulated and lower proportion of up-regulated targets in both miRNA and tRF groups of targets compared to all mRNAs. Although these proportions for mRNAs and tRF targets were generally comparable, we noted a bimodal distribution for tRF targets, whereas such bimodality was much less pronounced for miRNA targets ( Figure 7). Targets for both types of small RNAs show their most prominent peaks for low levels of down-regulation with age (these range from 0 to -5% and are possibly related to targeting relevant in other cellular contexts or false positives in target predictions). However, the proportion of tRF targets down-regulated in the range of 10.0–22.5%, and thus more likely to be relevant in the brain, is consistently higher compared to that of miRNA targets. Such (relatively low) level of change is not surprising, given that miRNAs are considered to be fine-tuning the transcriptional control by post-transcriptionally modulating the target transcript levels ^36^. The age-related decrease in the mRNA levels for tRF targets is generally more pronounced than that for miRNA targets.

### Finding missed tRNA genes

In our effort to identify every possible tRF present in rat brains, we took into account a union of all annotated rat tRNAs from two databases ( http://gtrnadb.ucsc.edu; http://trnadb.bioinf.uni-leipzig.de). Although the latter database is rather small compared to the former, we found that it contained a handful of rat tRNA genes (to which tRF fragments did map perfectly), which were missing from the UCSC database at the time of our first analysis. Upon subsequent checking, we found that most of the missing tRNA genes have been added correctly to the most recent update of the UCSC database (not including mitochondrial tRNAs). However, there is a tRNA gene (tdbD00000658-GluCTC; Table 2), which aligns perfectly to the rat genome (chr17:45,642,771-45,642,843 of rn6), and which is still absent in the latest version of the UCSC database. In our analysis, we detected tRFs from all nine sequencing libraries mapping to tdbD00000658-GluCTC sequence. Together with the fact that annotating tRNAs is not a typical priority in genome sequencing projects, our observations suggest that there are potentially other tRNA genes lacking annotation in the published genomes. However, such genes appear to be sources of detectable tRFs. Hence, analysis of tRFs can have an added value of revealing unannotated tRNA genes for multiple species.

## Discussion

In this study we characterized tRFs present in rat brains at three different time points, revealing that their abundance is dynamically regulated in the context of age. Previously, we have reported age-related changes in *D. melanogaster* tRFs ^23^. While only two time points have been considered in that paper, it has shown the changes related to the tRF loading to Argonaute proteins and thus very likely related to the function of the RISC complex. Here we observed two typical patterns of change in tRF levels. One was a monotonous increase with age, primarily seen in 3’ tRFs. Another was a lower abundance in mid-aged rat brains and higher abundance in young and old animals, mostly observed in 5’ tRFs. These patterns, together with the differences in fragment sizes, suggest distinct mechanisms of cleavage for the two types of fragments, which can potentially be attributed to the different roles for these two types of tRFs. In addition to the biogenesis pathways, tRFs originating from different ends of the tRNA molecule have also been shown to localize in different sub-cellular compartments. As pointed out by Kumar *et al.* ^21^, 5' tRFs were equally abundant in the nuclei and whole cell fraction of HeLa cell line ^37^, indicating primarily nuclear localization, which is consistent with large numbers of 5’ tRFs in HeLa cell nucleoli ^11^. On the contrary, 3’ tRFs showed an enrichment in the whole cell fraction, indicating their cytoplasmic localization (in agreement with Haussecker *et al.* ^12^). There has been evidence of miRNAs actively loaded to Argonaute proteins in an age-dependent manner in *D. melanogaster* ^20^. A very similar age-related loading pattern was also observed for *D. melanogaster* tRFs ^23^. This, along with extensive evidence that Argonaute proteins are not only acting in post-transcriptional silencing but are localized/imported to the nucleus, could imply additional unknown functions for tRFs within the nuclear compartments of the cell. Perhaps such functions are similar to those previously described for miRNAs, which have been shown to be associated with mRNA splicing and modulation of histone epigenetic modifications ^38, 39^. This is a focus of our ongoing research.

Although the mode of action for tRFs is yet to be elucidated, our results support the hypothesis that mammalian tRFs (at least, 3’ tRFs) can act in a very similar way to miRNAs in post-transcriptional gene silencing. We show here that they contain 7-mers, which match 3’ UTR regions of transcripts at much higher rates than expected by chance, similar to the seed sequences of miRNAs. Searching for conserved matches across vertebrate genomes, we found such seeds on either end of the tRF molecules, as has been the case with 12 *Drosophila* species ^23^. Previous studies have also detected both 5’ and 3’ seeds in different tRFs and changes in the seed sequence have been shown to affect the suppression of mRNA translation ^22, 29^. It is worth noting that in miRNAs, 3'-compensatory sites ^40^ and central pairing sites ^41^ have been reported in addition to the most prevalent 5’ seeds ^34, 35^; thus, finding seeds on both ends of tRFs is not unexpected. Non-traditional seed region location in miRNA is also consistent with the extensive results of Helwak *et al.* ^42^, who reported that more than half of the observed miRNA-mRNA interactions do not show traditional seed binding properties in HEK-293 cells. However, one cannot exclude other modes of action, for example, ribosome targeting ^5^. Additionally, tRFs have been reported to bind to oncogenic RNA-binding protein YBX1, displacing pro-oncogenic transcripts and acting as tumor suppressors ^43^.

Interestingly, for tRFs with clearly defined seed-like regions, we observed a significant and consistent enrichment for targeted genes, whose Gene Ontology terms were related to neuronal function and development. Again, this was in agreement with a functional enrichment seen in *Drosophila* tRF targets ^23^. However, in addition to these functions, rat brain tRFs also appeared to target transcription and splicing regulators, in parallel to earlier findings for rat brain miRNAs ^36^. Some of the genes were predicted to be targeted by more than one tRF ( Supplementary File 1), including well-known regulators of growth, such as PTEN or MAP3K1 (both targeted by three tRFs). These genes were seen down-regulated with age in the present study, consistent with their involvement in the developing nervous system. The highest number of tRFs (four) targeted the QK gene, whose human homolog has been implicated in oligodendrocyte-related gene expression abnormalities in schizophrenia ^44^.

Having identified potential targets of 3’ tRFs, we compared age-related changes in their transcript levels with the targets of upregulated miRNAs and observed small but significant down-regulation of such targets for both groups of small RNAs. However, tRFs appeared to have more of their targets down-regulated to a greater extent with age compared to those of miRNAs. Among such down-regulated tRF targets with a well-defined role in the nervous system, a netrin receptor, UNC5C, is related to axon guidance and neural development. A mutation in this gene has been associated with predisposition to Alzheimer's disease and has been shown to cause increased neuronal cell death in rodents ^45^. Cadherin genes, which are related to development and maintenance of functional structures in the central nervous system (reviewed in 46) were found in the present study to be targeted by tRFs (PCDH9). Fibroblast growth factor receptor-2 gene (FGFR2) was also found among the targets, suggesting that tRFs may affect key proteins involved in neural development, given that fibroblast growth factors are potent modulators of proliferation in the developing nervous system ^47^.

As is the case with miRNAs, different tRFs appeared to affect down-regulation of their targets to a different extent with age ( Table 3). The RISC pathway functions by repressing translation and by mRNA cleavage, and the exact balance of those mechanisms is not known. It has been speculated that degradation of repressed mRNAs by other mechanisms may be responsible for the observed decrease in their counts ^48^. It is also unclear if the miRNA and tRF levels determined by RNA-seq correlate with their actual functional levels in the RISC complexes, or if the tRF entry into the RISC system in mammals is guarded, as seen in the yeast *Schizosaccharomyces pombe* ^31^. Nevertheless, tRF targets appear to be more efficiently down-regulated compared to miRNA targets in aging rat brains ( Figure 7). These present findings await experimental validation and may be of relevance for human aging and neurodegeneration studies, given the comparable gross structure of the rat and human brains and the role of rat models in neurological research.

## Data availability

The data referenced by this article are under copyright with the following copyright statement: Copyright: © 2016 Karaiskos S and Grigoriev A

We have provided an archive with the data that should allow others to reproduce our results and figures presented in this paper. This archive is available via the Open Science Framework at https://osf.io/hz8en/, DOI: 10.17605/OSF.IO/HZ8EN ^49^.

It contains output .txt files produced in the course of this study, including the following: (1) A description of output files (readme.txt); (2) 9 *.tRNAs files, generated using Bowtie, which include all possible reads that mapped to tRNA genes; (3) 54 *.txt files, which are the output from the seed sequence identification pipeline.

The file format for the seed sequence identification pipeline output is as follows:

Column 1)    Gene name
Column 2)    7-mer location on the tRF molecule
Column 3)    Multiple Sequence alignment starting position
Column 4)    Multiple Sequence alignment ending position
Column 5)    3' UTR starting position
Column 6)    3' UTR ending position
Column 7)    Match type
Column 8)    Species with this site type (Taxonomy ID, see Table 1)

Additional data are available from public repositories as follows: small RNA sequencing libraries, European Nucleotide Archive (accession number, ERA365111); transcript levels in the cortex transcriptome, GEO data series (accession number, GSE34272); miRNA targets, Targetscan ( http://www.targetscan.org/cgi-bin/targetscan/data_download.cgi?db=mmu_71).

## Supplementary material

**Supplementary File 1.** List of predicted conserved tRF targets for the six tRFs shown in Figure 6.

.

**Supplementary File 2.** Gene Ontology enrichment analysis for the six tRFs shown in Figure 6.
