# Supplementary material for: Dynamics of tRNA fragments and their targets in aging mammalian brain
Source: F1000Res. 2016 Nov 24;5:ISCB Comm J-2758. [Version 1] doi: 10.12688/f1000research.10116.1 (PMC5224686; doi:10.12688/f1000research.10116.1)
Supplement: Peer review history

# Peer review: Referee response for version 1

**DOI:** 10.5256/f1000research.10897.r18880

**Published:** 2017-01-03

**Authors:** Laura Kamenetzky, Natalia Macchiaroli

**Affiliations:** 1 Institute of Medical Microbiology and Parasitology (IMPaM), Faculty of Medicine, University of Buenos Aires (UBA) and National Scientific and Technical Research Council (CONICET), Buenos Aires, Argentina

The manuscript adds important and relevant work on tRFs present in rat brains at three different time points. The methodology is well-written and able to be replicated. The results demonstrate that tRFs abundance is dynamically regulated in the context of age and that tRFs contain 7-mers which match 3’ UTR regions of relevant mRNAs. Also, the tRFs analysis have an added value revealing unannotated tRNA genes for multiple species.

 There are minor issues (indicated below):

- It would be interesting to add more detail of the criteria used to determine the expression level of the miRNAs that are compared with the tRFs. It may also be explained in more detailed how the authors selected the 5 miRNAs for comparisons.
- It would be interesting to show a table with the number of tRFs sites (single or multiple) per mRNA target since in the supplementary material it is difficult to see. Also targets regulated by distinct tRFs.
- The legend of Figure 4 mentions the yellow color that is absent in the figure.

We have read this submission. We believe that we have an appropriate level of expertise to confirm that it is of an acceptable scientific standard.

# Peer review: Referee response for version 1

**DOI:** 10.5256/f1000research.10897.r17976

**Published:** 2016-12-16

**Authors:** Tatiana V. Tatarinova

**Affiliations:** 1 Center for Personalized Medicine (CPM), Spatial Sciences Institute, Children's Hospital Los Angeles, University of Southern California , Los Angeles, CA, USA

The paper is well-written with appropriate title and adequate abstract. There are, however, several minor comments.

1. The authors mention in passing the results of Helwak et al. Would it be possible to validate the predicted seed regions using the data from the CLASH experiments?
2. The results appear to agree with their previous work in Drosophila, with the difference that Ago IP was used in the flies. This difference is not discussed in sufficient detail here and needs to be further elaborated.
3. Have the authors considered a “target” of the tRNA itself, from which a fragment is produced? After all, there is base complementarity in the stem for both 5’ and 3’ tRFs.
4. A recent paper in Science ^1^ implicated possible tRF matches in promoter regions. Have the authors considered these targets?

I have read this submission. I believe that I have an appropriate level of expertise to confirm that it is of an acceptable scientific standard.

## References

[1] ChenQYanMCaoZLiXZhangYShiJFengGHPengHZhangXZhangYQianJDuanEZhaiQZhouQ: Sperm tsRNAs contribute to intergenerational inheritance of an acquired metabolic disorder. *Science*.2016;351(6271) : 10.1126/science.aad7977 397-400 10.1126/science.aad7977 26721680
